# Supplementation of vitamin E as an addition to a commercial renal diet does not prolong survival of cats with chronic kidney disease

**DOI:** 10.1186/s12917-024-04176-8

**Published:** 2024-07-10

**Authors:** Martina Krofič Žel, Gabrijela Tavčar Kalcher, Tomaž Vovk, Bojana Žegura, Lara Lusa, Nataša Tozon, Alenka Nemec Svete

**Affiliations:** 1 Veterinary Faculty, Small Animal Clinic, University of Ljubljana, Gerbičeva 60, Ljubljana, 1000 Slovenia; 2 Veterinary Faculty, Institute of Hygiene and Pathology of Animal Nutrition, University of Ljubljana, Gerbičeva 60, Ljubljana, 1000 Slovenia; 3 Faculty of Pharmacy, Department of Biopharmaceutics and Pharmacokinetics, University of Ljubljana, Aškerčeva 7, Ljubljana, 1000 Slovenia; 4 Department of Genetic Toxicology and Cancer Biology, National Institute of Biology, Večna pot 111, Ljubljana, 1000 Slovenia; 5 Faculty of Mathematics, Natural Sciences and Information Technologies, Department of Mathematics, University of Primorska, Glagoljaška 8, Koper, 6000 Slovenia; 6 Institute for Biostatistics and Medical Informatics, Medical Faculty, University of Ljubljana, Ljubljana, 1000 Slovenia

**Keywords:** Chronic kidney disease, Oxidative stress, Vitamin E, Oxidative stress, Survival

## Abstract

**Background:**

The aim of this double-blind, placebo-controlled study was to investigate the effect of vitamin E supplementation as an addition to a commercial renal diet on survival time of cats with different stages of chronic kidney disease (CKD). In addition, we were interested whether vitamin E supplementation affects selected oxidative stress and clinical parameters. Thirty-four cats with CKD and 38 healthy cats were included in the study. Cats with CKD were classified according to the IRIS Guidelines; seven in IRIS stage 1, 15 in IRIS stage 2, five in IRIS stage 3 and seven in IRIS stage 4. Cats with CKD were treated according to IRIS Guidelines. Cats with CKD were randomly assigned to receive vitamin E (100 IU/cat/day) or placebo (mineral oil) for 24 weeks in addition to standard therapy. Plasma malondialdehyde (MDA) and protein carbonyl (PC) concentrations, DNA damage of peripheral lymphocytes and plasma vitamin E concentrations were measured at baseline and four, eight, 16 and 24 weeks thereafter. Routine laboratory analyses and assessment of clinical signs were performed at each visit.

**Results:**

Vitamin E supplementation had no effect on the survival time and did not reduce the severity of clinical signs. Before vitamin E supplementation, no significant differences in vitamin E, MDA and PC concentrations were found between healthy and CKD cats. However, plasma MDA concentration was statistically significantly higher (*p* = 0.043) in cats with early CKD (IRIS stages 1 and 2) than in cats with advanced CKD (IRIS stages 3 and 4). Additionally, DNA damage was statistically significantly higher in healthy cats (*p* ≤ 0.001) than in CKD cats. Plasma vitamin E concentrations increased statistically significantly in the vitamin E group compared to the placebo group four (*p* = 0.013) and eight (*p* = 0.017) weeks after the start of vitamin E supplementation. During the study and after 24 weeks of vitamin E supplementation, plasma MDA and PC concentrations and DNA damage remained similar to pre-supplementation levels in both the placebo and vitamin E groups.

**Conclusions:**

Vitamin E supplementation as an addition to standard therapy does not prolong survival in feline CKD.

**Supplementary Information:**

The online version contains supplementary material available at 10.1186/s12917-024-04176-8.

## Background

Chronic kidney disease (CKD) is one of the most common reasons for mortality in cats [1]. Tubular degeneration, interstitial inflammation, fibrosis, and glomerulosclerosis are the most common histopathological changes found in cats with CKD; the severity of the mentioned changes being greater in the later stages of the disease [2].

However, in human CKD patients, CKD is mostly a glomerular disease, where oxidative stress is recognized as an important factor in CKD progression, acting as a mediator of chronic inflammation [3–6], and causing oxidative damage to membrane and plasma lipids, proteins, and DNA [3, 4, 7–17].

Despite several reports, there is still little insight into the role of oxidative stress in the pathophysiology of feline CKD due to inconsistency in the results of published studies [18–27]. It has been suggested that antioxidant defence mechanisms are induced in feline CKD [19, 20] and that oxidative stress may be highest in the early stages of CKD in cats as indicated by the increased levels of plasma and urine F2-isprostanes [22, 23], a well-known and reliable biomarker of lipid peroxidation and thus oxidative stress [28–30]. In another study, no differences in oxidative stress parameters in CKD cats compared to healthy cats were found [21]. In contrast, the authors of a recent study reported that plasma concentrations of malondialdehyde (MDA), a known lipid peroxidation biomarker, and 8-hydroxy-2^’^-deoxy-guanosine (8-OHdG), a biomarker of oxidative DNA damage, increase with the severity of feline CKD [26].

The serum concentration of vitamin E, a lipid soluble antioxidant that is a highly potent free radical scavenger [7, 31] protecting lipid membranes from peroxidation, is decreased in human [14] and canine (measured in plasma) [32] CKD patients due to increased production of reactive oxygen species. A significant negative correlation was found between serum vitamin E and C and serum creatinine, serum urea and serum MDA in human CKD patients [7].

Vitamin E supplementation in haemodialysis patients has been reported to decrease the extent of lipid peroxidation, as indicated by decreased serum MDA concentration after supplementation, and to increase antioxidant enzyme activity, erythrocyte superoxide dismutase and catalase activities, and concentrations of vitamins E and C [13]. The results of the systematic review and meta-analysis on the effects of vitamin E supplementation on MDA in haemodialysis patients also support the use of vitamin E to reduce lipid peroxidation and thus oxidative stress in these patients [33].

In human CKD patients it has been reported that vitamin E supplementation improved anaemia and response to erythropoietin treatment [34] and decreased the incidence of cardiovascular complications and atherosclerosis [35]. In dialysis patients, a protective effect of vitamin E has been noted with respect to oxidative DNA damage [36]. Moreover, it has been suggested that vitamin E may slow down the progression of CKD as has been shown in animal models [37]. Although the SPACE study (Secondary Prevention with Antioxidants of Cardiovascular disease in End-stage renal disease) did not show a significant difference in overall mortality, a significant decrease in combined cardiovascular events was noted in the group of patients receiving vitamin E [38]. In contrast, it has even been suggested that vitamin E supplementation in human CKD patients was not recommended as it has been shown to have no apparent effect on the overall mortality [39].

It is still unclear whether antioxidant treatment in cats with CKD has the same effect on clinical status and survival as in human patients [38, 40]. In feline CKD patients, vitamin E supplementation was reported to have no effect on total antioxidant capacity, measured as copper ion reducing antioxidant capacity, or anaemia [41]. In another study, supplementation with a combination of antioxidants (vitamin C, vitamin E and β-carotene) resulted in a significant decrease in oxidative DNA damage [21].

Studies have reported that commercial renal diets containing vitamin E and other antioxidants in different concentrations can prolong survival in cats with CKD [42, 43]. However, there are no studies that have investigated the effect of antioxidant supplementation as an addition to standard diet and therapy on survival in cats with CKD. Therefore, the aim of this study was to investigate the effect of vitamin E supplementation on survival time of cats with CKD. In addition, selected oxidative stress parameters, including plasma concentrations of vitamin E, MDA and protein carbonyls (PC), a biomarker of oxidative damage to proteins, and percentage of tail DNA (descriptor for DNA break frequencies assessed by comet assay), a biomarker of the extent of DNA damage, and clinical parameters were assessed at baseline and during the supplementation period.

## Results

### Cats

Thirty-five cats with CKD and 44 healthy cats (altogether 79 cats) of both sexes were enrolled in this study. Seven cats were excluded due to underlying disease: six from the group of healthy and one with CKD. Finally, 34 CKD cats were included in this double-blind, placebo-controlled study (Tables 1 and 2). Healthy cats were statistically significantly younger (Table 1).

The CKD cats received vitamin E or placebo for 24 weeks and were followed up for 45 months.

**Table 1:** Demographic characteristics and selected laboratory findings of CKD cats and healthy cats before supplementation

	RI	Healthy cats (*n* = 38)	CKD group (*n* = 34)		
IRIS Stage	–	None	IRIS 1 (*n* = 7)	IRIS 2 (*n* = 15)	IRIS 3 + 4 (*n* = 12)
F/M	–	24 (3 intact)/14 (3 intact)	3/4	8/7 (1 intact)	6/6
Age (years)	–	3.0^a^ (1.0–6.0)	12.0 (7.5–13.0)	8.0 (5.0–11.5)	12.5 (9.0–14.5)
Body weight (kg)	–	4.2 (3.4–4.9)	4.6 (4.2–6.1)	4.3 (4.0–5.6)	3.4 (3.1–5.7)
Vitamin E/placebo	–	None	3/4	7/8	8/4
Urea (mmol/l)	2.5–9.6	9.5 (7.8–10.5)	9.6 (8.3–11.0)	9.8 (9.2–12.3)	36.7^b^ (24.0–50.9)
Creatinine (µmol/l)	70.7–159.0	129.6 (110.8–139.9)	135.2 (127.1–137.7)	176.3 (153.1–193.4)	491.9^b^ (295.7–690.4)
P (mmol/l)	1.45–2.62	–	1.40 (1.17–1.54)	1.41 (1.26–1.52)	2.64^b^ (1.78–3.69)
UPC (unitless)	˂0.20	–	0.13 (0.09–0.22)	0.20 (0.09–0.50)	0.45^c^ (0.24–0.92)
SBP (mm Hg)	˂150	–	125.0 (117.0–155.8)	131.0 (124.3–154.8)	162.0 (144.5–175.3)

Data are reported as median (interquartile range; 25–75th percentiles); RI = reference interval of the Laboratory of Small Animal Clinic, Veterinary Faculty Ljubljana; ^a^ statistically significant difference (*p* < 0.05) compared with groups of CKD patients (IRIS 1, IRIS 2, IRIS 3 + 4); ^b^ statistically significant difference (*p* < 0.05) compared with CKD stage 1 and CKD stage 2; ^c^ statistically significant difference (*p* < 0.05) compared with CKD stage 1; F = female cats; M = male cats^;^ P = inorganic phosphate; UPC = urine protein: creatinine ratio; SBP = systolic blood pressure

**Table 2:** Demographic characteristics and selected laboratory findings of CKD cats in the placebo/vitamin E group before supplementation

	RI	Placebo group (*n* = 16)	Vitamin E group (*n* = 18)
IRIS 1/2/3 + 4	–	4/8/4	3/7/8
F/M	–	8/8	9/9 (1 intact)
Age (years)	–	9.0 (5.0–13.0)	11.0 (6.0–14.0)
Body weight (kg)	–	4.3 (3.2–5.1)	4.6 (3.9–6.6)
Urea (mmol/l)	2.5–9.6	11.7 (9.3–19.8)	11.3 (9.3–35.1)
Creatinine (µmol/l)	70.7–159.0	173.9 (135.2–246.2)	193.0 (150.4–461.0)
P (mmol/l)	1.45–2.62	1.45 (1.16–1.62)	1.40 (1.32–2.61)
UPC (unitless)	˂0.20	0.24 (0.09–0.81)	0.24 (0.15–0.36)
SBP (mm Hg)	˂150	129.0 (123.3– 158.7)	155.0 (129.5–166.5)

Data are reported as median (interquartile range; 25–75th percentiles); RI = reference interval of the Laboratory of Small Animal Clinic, Veterinary Faculty Ljubljana; F = female cats; M = male cats; P = inorganic phosphate; UPC = urine protein: creatinine ratio; SBP = systolic blood pressure

During the study, three cats from the vitamin E group (staged IRIS 4 at inclusion) and four cats from the placebo group (stage at inclusion: two IRIS 4, one IRIS 3, and one IRIS 2) were euthanized due to severe signs of uraemia. The fluctuation of cases was not only due to the death of the animals, but due to the non-attendance of the owners (Table 3).

**Table 3:** Number of sampled cats on different occasions

Sampling time (weeks)	IRIS 1 + 2		IRIS 3 + 4	
	placebo	vitamin E	placebo	vitamin E
0	12	10	4	8
4	11	8	2	4
8	9	7	1	3
16	7	9	2	2
24	8	8	1	1

After the supplementation period of 24 weeks, the cats were monitored for up to 45 months.

During this time period, 4 more cats were euthanized and one died. One cat was euthanized due to severe signs of uraemia and four cats (two from the placebo group and two from the vitamin E group) due to non-renal causes: one due to lymphoma, one due to a car accident, two of unknown cause according to the owners.

During the study, there was no statistically significant difference in the clinical sign scores between the vitamin E and placebo groups at any stage of CKD (Table 4). Detailed scores are presented in Supplementary Table 1 (IRIS 1 + 2) and 2 (IRIS 3 + 4).

**Table 4:** Severity of clinical signs score during clinical study in cats with CKD IRIS 1 + 2 and IRIS CKD 3 + 4

Sampling time (week)		Score for IRIS 1 + 2		Score for IRIS 3 + 4		
		0‒4	5‒7	0‒4	5‒7	8‒10
0	Vitamin E	10	0	4	1	3
	Placebo	12	0	1	2	1
4	Vitamin E	8	0	2	2	0
	Placebo	11	0	2	0	0
8	Vitamin E	7	0	3	0	0
	Placebo	8	1	1	0	0
16	Vitamin E	9	0	2	0	0
	Placebo	7	0	2	0	0
24	Vitamin E	8	0	1	0	0
	Placebo	7	1	1	0	0

n = number of included cats; Score: 0‒4: mild clinical signs; 5‒7: moderate clinical signs; 8‒10: severe clinical signs; > 10: unacceptable clinical signs; euthanasia

### Survival time

**Fig. 1:**
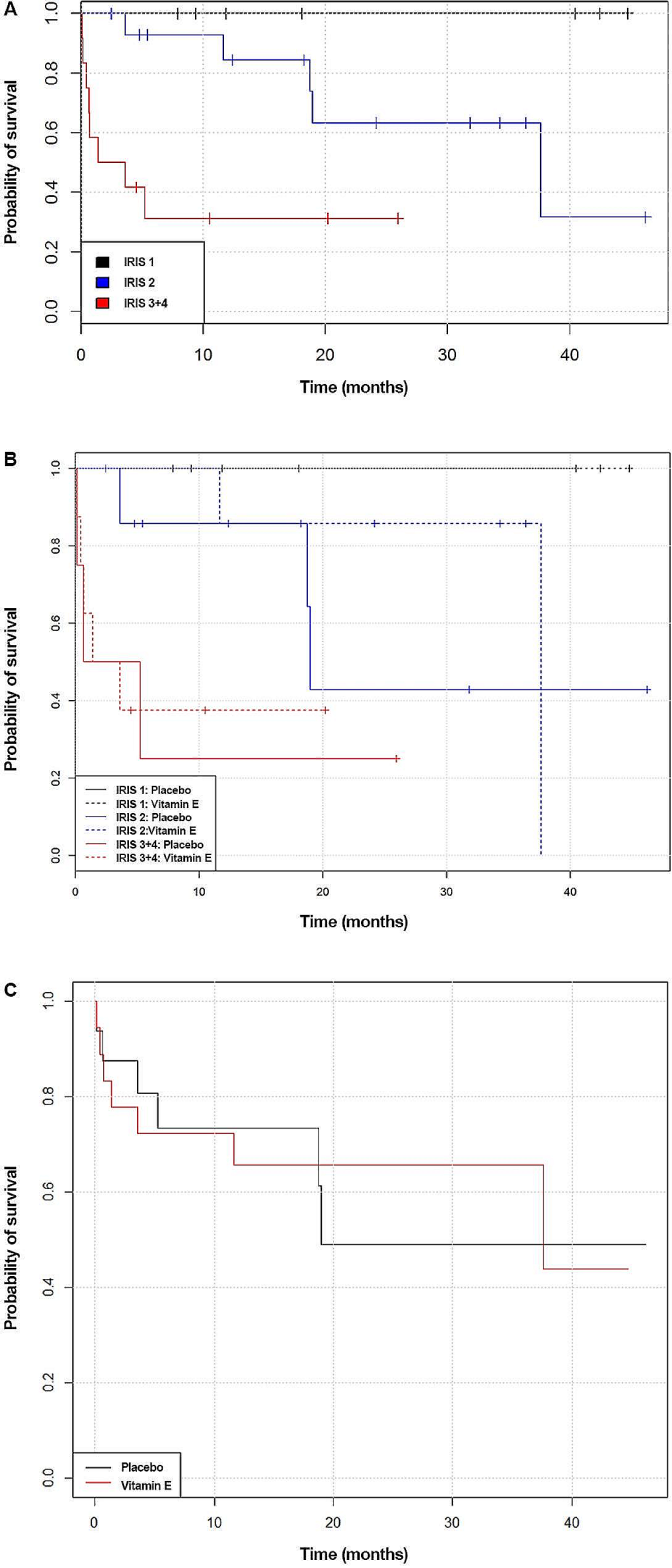
Survival of cats with CKD. **A** = cats treated with placebo or vitamin E are shown together according to the stage of the disease. **B** = cats treated with placebo or vitamin E are shown separately. **C** = overall comparison of survival of cats treated with placebo or vitamin E

The survival time was assessed from the beginning of the study at inclusion. Survival time was statistically significantly associated with the stage of CKD at inclusion (*p* = 0.001; Fig. 1A). Patients were followed up for 45 months. All cats in IRIS stage 1 survived during this period. The median survival time was 37.5 months in cats with IRIS stage 2 and 2.5 months in cats with IRIS stage 3 + 4.

There was no statistically significant difference in the survival time between cats in the vitamin E and placebo groups, regardless of CKD stage (Fig. 1B). In IRIS stage 2, the median survival time was 19 months in the placebo group and 37 months in the vitamin E group (*p* = 0.50). In IRIS stage 3 + 4, the median survival time was 2.95 months in the placebo group and 2.5 months in the vitamin E group (*p* = 0.80).

Overall comparison of the survival of all cats treated with placebo and all cats treated with vitamin E showed no significant difference (*p*>0.99) in survival curves (Fig. 1C). The median survival time was 19 months in the placebo group (95% CI: 18.7 months – NA) and 37.6 months in the treated group (95% CI: 11.7 months — NA).

### Oxidative stress parameters

#### Plasma vitamin E concentration

Before supplementation, there was no statistically significant difference in plasma vitamin E concentration between the vitamin E, control, and placebo groups (data not shown). Plasma vitamin E concentrations and lipid-standardized plasma vitamin E concentration (vitamin E (LS)) statistically significantly increased in the vitamin E group compared to placebo group four (*p* = 0.013 (vitamin E); *p* = 0.001 (vitamin E (LS)) and eight (*p* = 0.017 (vitamin E); *p* = 0.007 (vitamin E (LS)) weeks after the start of vitamin E supplementation (Table 5).

**Table 5:** Plasma vitamin E concentration and lipid-standardized vitamin E (vitamin E (LS)) during the study

Sampling time (week)	Placebo group Vitamin E (µmol/L)	Vitamin E group Vitamin E (µmol/L)	Placebo group Vitamin E (LS) × 10^− 3^ (unitless)	Vitamin E group Vitamin E (LS) × 10^− 3^ (unitless)
0	35.40 (26.20–45.18) *n* = 15	38.00 (30.70–52.00) *n* = 17	5.67 (4.56–7.58) *n* = 15	6.81 (5.8–8.69) *n* = 17
4	38.90 * (30.70–52.00) *n* = 12	65.30 (45.23–74.18) *n* = 11	6.61* (6.17–7.57) *n* = 12	10.12 (8.44–11.86) *n* = 11
8	38.30* (35.40–46.50) *n* = 10	68.95 (43.70–98.90) *n* = 10	6.28* (4.78–7.31) *n* = 10	11.42 (7.31–14.52) *n* = 10
16	35.90 (27.95–45.58) *n* = 9	42.20 (38.70–57.00) *n* = 11	7.05 (5.82–7.59) *n* = 9	7.79 (6.98–9.75) *n* = 11
24	38.20 (28.38–47.73) *n* = 9	68.40 (42.28–83.90) *n* = 9	7.03 (5.42–7.63) *n* = 9	9.59 (6.36–10.53) *n* = 9

Data are reported as median (interquartile range; 25–75th percentiles); * *p* < 0.05; n = number of sampled cats. Sample sizes differ between sampling times due to deaths of cats and because not all of the cats were presented by the owners on each sampling occasion (incompliance of the owners)

#### Plasma malondialdehyde and plasma protein carbonyl concentrations

There was no statistically significant difference in baseline plasma MDA or PC concentrations between cats with CKD and healthy cats. The baseline plasma MDA concentration in healthy cats was 3.50 (2.70–4.00) µmol/L and 2.90 (2.50–3.95) µmol/L in cats with CKD, while the baseline plasma PC concentration in healthy cats was 25.8 (20.6–34.3) nmol/mL and 31.4 (26.1–39.0) nmol/mL in cats with CKD.

However, plasma MDA concentration was statistically significantly higher (*p* = 0.043) in cats with CKD IRIS 1 and 2 (3.74 (3.39–4.22) µmol/L) than in cats with advanced stages of CKD (3 and 4), where the plasma MDA concentration was 2.76 (2.65–3.14) µmol/L. Plasma MDA and PC concentrations in the placebo group and in the vitamin E group before supplementation and after 4, 8, and 24 weeks of supplementation are presented in Table 6.

**Table 6:** Plasma MDA and PC concentrations in the placebo and vitamin E groups during the study

Sampling time (week)	PC (nmol/mL)		MDA (µmol/L)		
	Placebo group	Vitamin E group		Placebo group	Vitamin E group
0	30.70 (26.55–40.60) (*n* = 15)	31.60 (25.62–36.98) (*n* = 17)		3.52 (3.00–4.03) (*n* = 12)	3.29 (2.73–3.91) (*n* = 12)
4	31.90 (24.95–38.00) (*n* = 11)	31.25 (27.98–37.05) (*n* = 10)		3.50 (2.62–3.71) (*n* = 9)	3.69 (3.23–4.56) (*n* = 10)
8	31.55 (26.52–32.98) (*n* = 15)	30.10 (26.73–36.38) (*n* = 17)		3.93 (3.66–4.39) (*n* = 8)	4.16 (3.48–4.60) (*n* = 9)
24	37.75 (29.56–42.13) (*n* = 8)	29.23 (24.27–39.74) (*n* = 9)		3.97 (3.10–4.79) (*n* = 7)	3.65 (2.24–4.09) (*n* = 6)

Data are reported as median (interquartile range; 25–75th percentiles); MDA = plasma malondialdehyde concentration; n = number of sampled cats; PC = plasma protein carbonyl concentration

### DNA damage

**Fig. 2:**
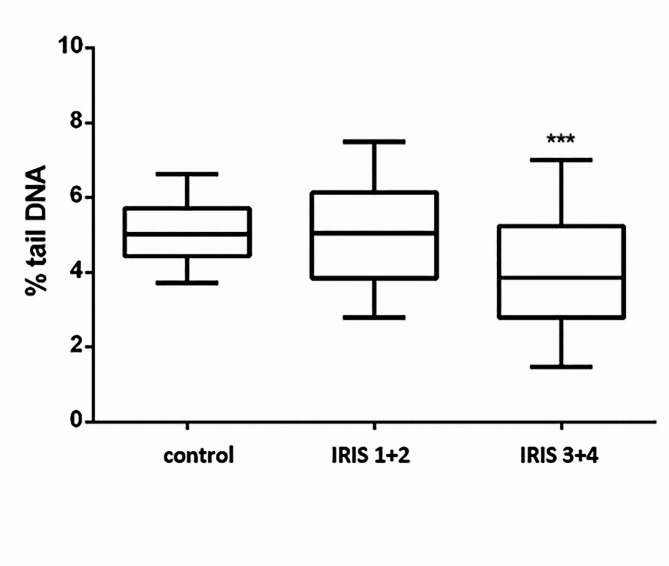
Percentage of tail DNA in cats with CKD and in the control group before supplementation. The results are presented in box plots. The asterisks (***) denote statistically significant difference (Kruskal-Wallis nonparametric test and Dunn’s multiple comparison test) between control and CKD group (*** *p* ≤ 0.001); n (control) = 38; n (IRIS 1 + 2) = 18; n (IRIS 3 + 4) = 8

Cats in the group of healthy had a statistically significantly higher percentage of tail DNA compared to cats with IRIS stage 3 + 4 before supplementation (*p* ≤ 0.001, Fig. 2). Although cats in stage 3 + 4 had lower percentage of tail DNA than cats in IRIS 1 + 2 the difference was not statistically significant. Percentage of tail DNA in the group of cats of all IRIS stages receiving placebo or vitamin E before supplementation and after 4, 8 and 24 weeks of supplementation are presented in Table 7.

**Table 7:** Percentage of tail DNA in the placebo and vitamin E groups during the study

Sampling time (week)	% tail DNA	
	Placebo group	Vitamin E group
0	2.215 (0.120–7.612) (*n* = 11)	2.082 (0.157–6.990) (*n* = 12)
4	3.140 (0.318–8.667) (*n* = 5)	3.023 (0.152–8.354) (*n* = 7)
8	2.273 (0.100–7.378) (*n* = 4)	2.808 (0.152–8.886) (*n* = 7)
24	2.321 (0.145–6.491) (*n* = 5)	2.120 (0.144–5.733) (*n* = 5)

Data are reported as median (interquartile range; 25–75th percentiles); n = number of sampled cats

## Discussion

The present double-blind, placebo-controlled study tested the effect of vitamin E supplementation on the survival of cats with CKD. To the authors’ knowledge, this is the first study dealing with this topic in cats.

The results showed no effect of vitamin E supplementation on the survival time nor on the severity of clinical signs. Survival time was statistically significantly dependent on the stage of CKD at inclusion (before supplementation), which is in accordance with previously published data [42–46]. According to current knowledge, the renal diet is thus the only therapy proven to prolong survival in cats and dogs with CKD [42, 47, 48].

Plasma vitamin E concentrations in cats with CKD included in our study were consistent with previously published data [21]. A statistically significant difference in plasma vitamin E concentration between the placebo and vitamin E groups was found four and eight weeks after the start of supplementation when all CKD cats at the same sampling occasion were grouped together. The same results were obtained with lipid-standardized vitamin E. However, vitamin E concentration and lipid-standardized vitamin E did not differ statistically significantly between the placebo and vitamin E groups after 24 weeks of supplementation, which could be due to the distribution of vitamin E in adipose tissue [49, 50], increased excretion [50], or lower number of cats remaining in the study. Besides, client owned cats were included where owner compliance regarding vitamin E supplementation could not be controlled as in a standardized environment.

In contrast to a previous report on CKD dogs [32], none of the cats with CKD included in our study exhibited vitamin E deficiency; their plasma vitamin E concentrations were near or even above the upper limit of the reference interval [51].

At least 540 IU of vitamin E per kilogram of dry matter of food is required to lower the concentration of serum MDA and 4-hydroxynonenal of healthy cats [52]. Various dosages of vitamin E as an antioxidant are recommended: 30 IU/cat q24h [53, 54]; 10‒15 IU/cat q24h [49]; 10‒20 IU/kg q12h [54]. The cats in our study were fed a commercial diet with a vitamin E containing 750 IU/kg dry matter, which corresponds to a daily intake of 30 IU/cat q24h. In addition, each cat in the vitamin E group received 100 IU vitamin E per day or 30 IU/kg q24h. As oxidative stress was assumed to be present in cats with CKD, the chosen dose was in the upper range of the recommendations.

In a previous study in CKD cats, 30 IU of vitamin E daily per cat had no effect on measures of oxidative stress or anaemia [41]. The authors suggested that the vitamin E dose may have been too low to be effective, although the vitamin E was not measured in the study [41]. Since there was a statistically significant increase in plasma vitamin E concentration in our study, we can assume that the selected vitamin E dose was appropriate.

As already mentioned, high doses of vitamin E were used in the present study. To the authors’ knowledge, there are currently no studies that would evaluate high dose vitamin E therapies for long time periods in cats. To the authors’ knowledge, only two studies [21, 41] investigated vitamin E supplementation in CKD cats. In one study, a supplementation period lasted for 4 weeks [21] and in the other study, the supplementation period lasted for 3 months [41]. The cats in the present study already received a kidney diet that was supplemented with vitamin E, and there could be a risk of possible overdosing if the supplementation period was longer.

As the metabolites of vitamin E are excreted in urine and the glomerular filtration rate is lowered in human CKD patients, the excretion of vitamin E metabolites may be impaired [55], which may also be true for cats. Furthermore, in human patients [55], plasma concentration of vitamin E may reach a plateau, so plasma vitamin E concentration may not be used as a measure of controlling the dose of vitamin E. In the present study, client-owned cats were used, so there was no possibility to monitor the urinary excretion of vitamin E.

Moreover, some deaths appeared in IRIS stage 3 and 4 during the supplementation period. This was in accordance with the shorter survival expectance and did not significantly differ from the placebo group. On the other hand, the early stages had to be evaluated during a longer period in order to rule out possible long term side effects of the supplementation. Therefore, the supplementation period was set at 24 weeks and the observation period lasted for several months. Before supplementation, no statistically significant difference in the degree of lipid peroxidation (MDA) and protein carbonylation (PC) was observed between cats with CKD and the group of healthy. In contrast to our results, increased levels of markers of lipid peroxidation (MDA; plasma and/or urinary F2-isoprostanes) [18, 22, 23, 26], and oxidative damage to proteins (advanced oxidation protein products) [18] and oxidative damage to DNA (8-OHdG) [26] have been reported in CKD cats compared with healthy cats. Similar results were obtained in human CKD patients [56, 57]. The discrepancy between our results and those previously reported in CKD cats might be due to the difference in type and methods for determination of oxidative stress parameters and the fact that in our study cats of all stages were included in CKD group, whereas in other studies, cats in IRIS stages 1–2 [23] and cats in IRIS stages 2–4 [21] were included. Furthermore, according to the IRIS classification, cats with different aetiologies of CKD are included in the same IRIS stage; different aetiologies could result in different levels of oxidative damage to lipids, proteins, and DNA, especially when dealing with tubular vs. glomerular processes. Further studies are warranted to examine whether various causes of CKD might result in different oxidative damage.

After 24 weeks of vitamin E supplementation, plasma MDA and PC concentrations remained similar to pre-supplementation levels. It was reported previously that the supplementation of a combination of antioxidants (vitamin E, vitamin C and β-carotene) in CKD cats resulted in a statistically significant decrease in oxidative DNA damage and had no statistically significant effect on the concentration of MDA [21]. The mentioned study [21] is difficult to compare with the present one because the effect of a combination of antioxidants was tested and different methods were used to determine oxidative DNA damage and MDA concentration.

In our study, plasma MDA concentration was statistically significantly higher in cats with early CKD (IRIS stages 1 and 2) than in cats with advanced stages of CKD. Similarly, statistically significantly increased level of the lipid peroxidation markers, urinary [22, 23] and plasma [23] F2-isoprostanes, were found in early CKD. In another study, no significant difference in plasma MDA concentration between cats in IRIS stage 2 and IRIS stage 3–4 was reported [26]. Our results suggest that there may be an induction of antioxidant defence mechanisms that prevent lipid peroxidation in later stages of CKD, as suggested by previously published studies [19, 20, 22].

Unexpectedly, DNA damage was statistically significantly higher in cats from the control group compared with cats with CKD. The comet assay is a very sensitive method, which, in addition to DNA single and double strand breaks, detects DNA breaks associated with incomplete excision repair [58, 59]. Therefore, the reason for the greater DNA damage in healthy cats could be due to the greater DNA repair capacity of healthy cats. Besides, polymorphisms and individual differences between subjects must also be considered. In contrast, no significant differences in DNA damage between healthy cats and those with CKD were found [21]. However, studies in human CKD patients showed higher levels of DNA damage that increased with disease progression [11, 60]. During the study and after 24 weeks of vitamin E supplementation, the percentage of tail DNA in both the placebo and vitamin E groups remained similar to that before supplementation.

The main limitation of the study is a small number of subjects studied, especially in IRIS stages 3 and 4 where the survival time is the shortest [44]. Small sample size made impossible to study the effects of vitamin E supplementation on selected oxidative stress parameters within each CKD stage.

Another limitation is that the mortality of cats in IRIS 3 and 4 already occurred during the vitamin E supplementation period, as deceased cats were included in the survival analysis, which can introduce bias and impact the results. However, the exclusion of deceased cats in the survival analysis could lead to an overestimation of the survival and too optimistic conclusions. Additional limitation of the study is a relatively short supplementation time. The last limitation of the study is the measurement of a single biomarker for lipid peroxidation (MDA), protein damage (PC), and DNA damage (percentage tail DNA). Selected biomarkers provide valuable insights, but a more comprehensive approach is warranted.

## Conclusions

Vitamin E supplementation for 24 weeks as an addition to standard therapy including a commercially produced therapeutic diet formulated for management of CKD did not significantly prolong the survival of cats across different CKD stages during a 45-week monitoring period.

Before the vitamin E supplementation, no significant differences were observed in selected oxidative stress parameters, including plasma concentrations of vitamin E, MDA, and PC between healthy cats and those with different stages of CKD. Surprisingly, CKD cats exhibited a significantly lower percentage of tail DNA compared to healthy cats. These intriguing results suggest the need for more extensive investigations into an expanded range of biomarkers related to oxidative damage to lipids, proteins, and DNA in a larger cohort of CKD cats, stratified by individual stages.

Extended supplementation periods should be explored, particularly in CKD patients who do not receive a vitamin E-rich diet. These additional studies will provide valuable insights into the impact of supplementation of vitamin E on oxidative stress parameters and overall management strategies of CKD cats.

## Materials and methods

### Cats

#### Inclusion criteria

CKD cats were divided into a placebo group and a vitamin E group that were staged according to IRIS Guidelines [61]. In addition, in order to measure and compare the DNA damage of peripheral lymphocytes in the cats with CKD, 38 healthy control cats were included.

Cats were considered healthy based on their history, physical examination, and the results of complete blood count and white blood cell differential count, and biochemical analysis.

Seven cats were staged in IRIS stage 1, 15 in IRIS stage 2, five in IRIS stage 3 and seven in IRIS stage 4.

All owners signed an informed consent form. All procedures were in accordance with relevant Slovenian governmental regulations (Animal Protection Act, Official Gazette of the Republic of Slovenia, No. 43/2007).

#### Exclusion criteria

The cats with acute kidney injury (the condition in which there is an abrupt reduction in renal function) [62, 63], prerenal (reduced renal perfusion resulting in reduced glomerular filtration rate) [64] or postrenal azotaemia (obstructive nephropathy) [64], nephropathy of toxic or infectious (bacterial pyelonephritis) [64, 65] origin within the past 28 days, urinary tract obstruction, acute systemic inflammation, liver disease, chronic heart failure, cancer, or those with clinical signs of feline leukaemia or feline immunodeficiency were excluded from the study.

### Treatment and vitamin E supplementation

The cats with CKD were treated according to IRIS Treatment Recommendations [66]. Correction of dehydration (if diagnosed at regular check-up visits) with subcutaneous or intravenous fluids (Hartmann solution Braun; B. Braun), correction of systemic hypertension (amlodipine 0.625 mg/cat q24h PO, Amlopin; Lek), phosphate binders in case of hyperphosphatemia (calcium carbonate and chitosan 1 g/cat q12h PO; Ipakitine; Vetoquinol), and antiemetics (maropitant 1 mg/kg q24h SC or 2 mg/kg q24h PO; Cerenia; Zoetis) if indicated. In addition, H_2_ receptors antagonists (ranitidine 3 mg/kg q12h PO or IM; Ranital; Lek) were administered if indicated. During the follow-up period as well as if exhibiting any worsening of the clinical signs, the cats were carefully examined and biochemistry, haematology, and diagnostic imaging were performed in order to exclude any concomitant disease, including acute on chronic kidney disease [67].

During the study, all CKD cats received the same commercial renal diet containing 750 IU of vitamin E per kg dry matter (Renal Support; Royal Canin) and 100 IU vitamin E/cat q24h PO in the form of d-alpha-tocopheryl acetate dissolved in vegetable oils (soya, corn, wheat germ, sesame, and lemon oil) (E-oil; Natural Wealth) or placebo (mineral oil; Paraffinum Liquidum, Pharmachem). Vitamin E or placebo respectively were applied to food in the form of oil drops immediately before consummation. The cats were randomly divided into the placebo or vitamin E group; simple randomization method with sequentially numbered, sealed envelopes, was used to group the patients [68]. The samples were given a consecutive number and the analyst did not know which sample belonged to the animal receiving placebo or vitamin E. The owners received either vitamin E or placebo in unmarked plastic flasks. The supplementation period lasted for 24 weeks, and the patients were followed up for 45 months.

### Blood and urine sampling and processing

The selected haematological, biochemical and oxidative stress parameters (plasma MDA and PC concentrations, and vitamin E concentrations, DNA damage of peripheral lymphocytes) were measured before supplementation (at inclusion) and 4, 8, 16, and 24 weeks after vitamin E supplementation. On each occasion, typical clinical signs associated with the development of CKD [69] were assessed. Each clinical sign was evaluated numerically, and the sum was recorded for each check-up (Supplementary Table 2).

In cats in the control group, selected haematological and biochemical parameters and DNA damage of peripheral lymphocytes were measured on one occasion. Furthermore, plasma MDA and PC concentrations were measured on one occasion in some healthy cats.

Blood samples were collected from the jugular vein and 1 mL was transferred to serum separator tubes (Vacuette; Greiner Bio-One) for the determination of serum biochemical profiles. The tubes were centrifuged at 1300 × g for 10 min at room temperature to separate the serum. Serum samples were analysed on the same day of collection.

0.5 mL of blood was transferred into EDTA–containing tube (BD Microtainer Tubes; Dickinson and Company) for haematological analysis and comet assay.

2 mL of blood were transferred into EDTA–containing tube (Vacuette; Greiner Bio-One) for the determination of plasma MDA and PC concentrations.

2 mL of blood were transferred into lithium heparin–containing tubes (Vacuette; Greiner Bio-One) for the determination of plasma vitamin E concentrations. All samples were immediately centrifuged at 1500 × g for 15 min at 4 °C. Plasma was separated and immediately frozen at − 80 °C until the analysis.

Urine samples were collected by cystocentesis and analysed within 1 to 2 h.

### Systolic blood pressure measurement

In all cats diagnosed with CKD, systolic blood pressure (SBP) was measured at each check-up with High Definition Oscillometry (HDO; S + B MedVet) in the lateral recumbency on the front right leg. Five successive measurements were performed, and the mean value was calculated. If hypertension was diagnosed (mean SBP over 160 mmHg), the cats were treated according to the previously described protocol.

### Analytical methods

#### Biochemical and haematological analyses

Biochemical profiles, with the exception of electrolytes, were determined with an automated biochemistry analyser (RX Daytona; Randox). Electrolytes were determined using an electrolyte analyser (Ilyte; Instrumentation Laboratory). Haematological analyses were performed with an automated laser haematology analyser (ADVIA 120; Siemens Healthcare Diagnostics). Immediately after completion of the haematology analysis, samples were stored at 4 °C for up to three hours until slides were prepared for the comet assay.

#### Urinalysis

Urinalysis included measurement of specific gravity with a refractometer, use of a standard multitest urine dipstick (Multistix 10SG; Siemens Healthcare Diagnostics) and microscopic examination of the urine sediment. Urine samples were centrifuged at 800 × g for 10 min at room temperature. Urine supernatants were used for determination of protein and creatinine concentrations to calculate the urine protein: creatinine ratio (UPC). Protein and creatinine concentrations were measured with an automated biochemistry analyser (RX Daytona; Randox) using the pyrogallol red and picric acid methods, respectively.

#### Determination of vitamin E and malondialdehyde concentrations

Plasma vitamin E concentration was determined by High Performance Liquid Chromatography with fluorescence detector (Alliance HPLC System 2695; Waters) using the method described by Zhao et al. [70] and Sivertsen et al. [71]. The inter- and intra-day coefficient of variation was 7% and 18%, respectively. Vitamin E concentration was standardized to the sum of serum cholesterol and triglyceride concentration [72, 73].

Total plasma MDA concentration was determined using the derivatization method by Czauderna et al. [74]. MDA was derivatized with 2,4-dinitrophenylhydrazine (2,4-DNPH) to a pyrazole derivative. The MDA derivative was analysed using an Agilent 1290 Infinity HPLC coupled to an Agilent 6460 Triple-Quadrupole mass spectrometer equipped with a JetStream electro-spray-ionization source (Agilent Technologies, Inc.). The inter- and intra-day coefficient of variation was 13.4% and 12.7%, respectively.

#### Determination of protein carbonyl concentrations

Protein carbonyl concentrations were determined using a commercially available kit (Protein carbonyl assay kit; Cayman Chemical) according to the manufacturer’s instructions. The method is based on the procedure developed by Levine et al. [75].

### Comet assay

The degree of DNA damage was measured by alkaline comet assay as described by Tice et al. [58] and Møller et al. [59] with minor modifications. Analysis was performed by a fluorescence microscope (Nikon Eclipse E 800; Nikon) connected through a black and white camera to an image analysis system (Comet Assay IV; Perceptive Instruments Ltd.). The % of tail DNA was used to measure the level of DNA damage and a total of 150 randomly captured nuclei were examined from each individual.

### Statistical analysis

Descriptive statistics were calculated for all haematological, biochemical, urinary, and oxidative stress parameters and for SBP (data not shown). Data were analysed using the computer programme R (R Core Team, 2015), GraphPad Prism and SigmaPlot 11.0 software. The values of the selected parameters (age, weight, biochemical parameters, SBP, plasma MDA, vitamin E and PC concentrations, percentage tail DNA and severity of clinical signs) were compared between the group of healthy and CKD cats and between the groups of CKD cats. Nonparametric Mann-Whitney test was used for comparison of parameters between two groups (healthy versus all CKD; placebo versus vitamin E; IRIS stage 1 + 2 versus IRIS stage 3 + 4). Nonparametric Kruskal-Wallis and Dunn’s multiple comparison post hoc test was used for the comparison of selected parameters among three and four groups of cats (control versus IRIS stage 1 versus IRIS stage 2 versus IRIS stage 3 + 4; control versus vitamin E versus placebo; control versus IRIS 1 + 2 versus IRIS 3 + 4). Because of the small number of cats in IRIS stage 3 and 4, these two stages were combined into one group.

Due to the small number of cats in the placebo and vitamin E groups that completed the supplementation study, the statistical difference in oxidative stress parameters before and at the end of supplementation (placebo or vitamin E) was not calculated.

In assessing the degree of DNA damage, cats with CKD IRIS stage 1 and 2 and cats with CKD IRIS stage 3 and 4 were grouped together and data were compared between the two groups, as well as in comparison to control group of healthy cats.

Survival time was assessed from the beginning of the study. Survival rate was calculated using the Kaplan–Meier analysis. Confidence intervals were reported only for the overall comparison of survival of cats of all IRIS stages in the placebo group and in the vitamin E group. We have not reported the confidence intervals for the comparison of survival between other cat groups as they are too wide due to the small cat groups.

A value of *P* ≤ 0.05 was considered statistically significant (*).

### Electronic supplementary material

Below is the link to the electronic supplementary material.

Supplementary Material 1

Supplementary Material 2

Supplementary Material 3

## Data Availability

All data are available from the corresponding author on reasonable request.
