# Supplementary material for: Supplementation of vitamin E as an addition to a commercial renal diet does not prolong survival of cats with chronic kidney disease
Source: BMC Vet Res. 2024 Jul 10;20:308. doi: 10.1186/s12917-024-04176-8 (PMC11234628; doi:10.1186/s12917-024-04176-8)
Supplement: Supplementary file 1 — Supplementary Material 1 [file 12917_2024_4176_MOESM1_ESM.docx]

Supplementary table1: Severity of clinical signs assessment during clinical study in cats with CKD IRIS 1+2

| Occasion | | Alertness (assessed by the owner) | | | Body weight loss | | | | Dehydration | | | Appetite | | Nausea, vomiting | | | Ulcerations in  the oral cavity | Score | | |
| --- | --- | --- | --- | --- | --- | --- | --- | --- | --- | --- | --- | --- | --- | --- | --- | --- | --- | --- | --- | --- |
|  |  | Very alert | Alert | Moderately responsive | | ≤ 5 % | 5‒10 % | 10‒15 % | ≤ 5 % | 5‒10 % | ≥ 10 % | Normal | Decreased | Not observed | Sporadic | Often |  | 0‒4 | 5‒7 |  |
| 1 | Vitamin E  (n = 10) | **9** | **1** | **0** | | **10** | **0** | **0** | **10** | **0** | **0** | **9** | **1** | **10** | **0** | **0** | **0** | **10** | **0** |  |
|  | Placebo  (n = 12) | **10** | **2** | **0** | | **12** | **0** | **0** | **12** | **0** | **0** | **10** | **2** | **7** | **5** | **0** | **0** | **12** | **0** |  |
| 2 | Vitamin E  (n = 8) | **8** | **0** | **0** | | **7** | **1** | **0** | **8** | **0** | **0** | **8** | **0** | **8** | **0** | **0** | **0** | **8** | **0** |  |
|  | Placebo  (n = 11) | **9** | **2** | **0** | | **7** | **4** | **0** | **11** | **0** | **0** | **8** | **3** | **11** | **0** | **0** | **0** | **11** | **0** |  |
| 3 | Vitamin E  (n = 7) | **6** | **1** | **0** | | **7** | **0** | **0** | **7** | **0** | **0** | **6** | **1** | **6** | **1** | **0** | **1** | **7** | **0** |  |
|  | Placebo  (n = 9) | **6** | **3** | **0** | | **9** | **0** | **0** | **8** | **0** | **1** | **8** | **1** | **5** | **4** | **0** | **0** | **8** | **1** |  |
| 4 | Vitamin E  (n = 9) | **9** | **0** | **0** | | **8** | **1** | **0** | **8** | **1** | **0** | **8** | **1** | **9** | **0** | **0** | **0** | **9** | **0** |  |
|  | Placebo  (n = 7) | **7** | **0** | **0** | | **6** | **1** | **0** | **7** | **0** | **0** | **7** | **0** | **5** | **2** | **0** | **0** | **7** | **0** |  |
| 5 | Vitamin E  (n = 8) | **7** | **1** | **0** | | **7** | **1** | **0** | **7** | **1** | **0** | **7** | **1** | **7** | **1** | **0** | **1** | **8** | **0** |  |
|  | Placebo  (n = 8) | **7** | **0** | **1** | | **6** | **1** | **1** | **7** | **1** | **0** | **7** | **1** | **7** | **0** | **1** | **1** | **7** | **1** |  |

Legend: n number of included cats; Score: 0‒4 mild clinical signs; 5‒7: moderate clinical signs; 8‒10: severe clinical signs; > 10: unacceptable clinical signs; euthanasia
