# Supplementary material for: Supplementation of vitamin E as an addition to a commercial renal diet does not prolong survival of cats with chronic kidney disease
Source: BMC Vet Res. 2024 Jul 10;20:308. doi: 10.1186/s12917-024-04176-8 (PMC11234628; doi:10.1186/s12917-024-04176-8)
Supplement: Supplementary file 2 — Supplementary Material 2 [file 12917_2024_4176_MOESM2_ESM.docx]

Supplementary Table 2: Severity of clinical signs assessment during clinical study in cats with CKD IRIS 3+4

| Occasion | | Alertness (assessed by the owner) | | | Body weight loss | | | | | Dehydration | | | Appetite | | | Nausea, vomiting | | | Ulcerations in the oral cavity | Score | | |
| --- | --- | --- | --- | --- | --- | --- | --- | --- | --- | --- | --- | --- | --- | --- | --- | --- | --- | --- | --- | --- | --- | --- |
|  |  | Very alert | Alert | Moderately responsive | Non-responsive | ≤ 5 % | 5 ‒ 10 % | 10 ‒ 15 % | ≥ 15 % | ≤ 5 % | 5‒10 % | ≥ 10 % | Normal | Decreased | Anorexia | Not observed | Sporadic | Often |  | 0‒4 | 5‒7 | 8‒10 |
| 1 | Vitamin E  (n = 8) | **2** | **3** | **2** | **1** | **3** | **3** | **2** | **0** | **4** | **4** | **0** | **2** | **3** | **3** | **2** | **2** | **4** | **0** | **4** | **1** | **3** |
|  | Placebo  (n = 4) | **1** | **0** | **3** | **0** | **1** | **2** | **1** | **0** | **2** | **2** | **0** | **1** | **2** | **1** | **1** | **2** | **1** | **1** | **1** | **2** | **1** |
| 2 | Vitamin E  (n = 4) | **1** | **3** | **0** | **0** | **1** | **0** | **2** | **1** | **3** | **1** | **0** | **1** | **3** | **0** | **3** | **0** | **1** | **0** | **2** | **2** | **0** |
|  | Placebo  (n = 2) | **2** | **0** | **0** | **0** | **2** | **0** | **0** | **0** | **2** | **0** | **0** | **2** | **0** | **0** | **1** | **1** | **0** | **0** | **2** | **0** | **0** |
| 3 | Vitamin E  (n = 3) | **3** | **0** | **0** | **0** | **1** | **2** | **0** | **0** | **3** | **0** | **0** | **1** | **2** | **0** | **3** | **0** | **0** | **0** | **3** | **0** | **0** |
|  | Placebo  (n = 1) | **1** | **0** | **0** | **0** | **1** | **0** | **0** | **0** | **1** | **0** | **0** | **1** | **0** | **0** | **1** | **0** | **0** | **0** | **1** | **0** | **0** |
| 4 | Vitamin E  (n = 2) | **1** | **1** | **0** | **0** | **2** | **0** | **0** | **0** | **2** | **0** | **0** | **1** | **0** | **1** | **1** | **1** | **0** | **0** | **2** | **0** | **0** |
|  | Placebo  (n = 2) | **2** | **0** | **0** | **0** | **1** | **0** | **1** | **0** | **2** | **0** | **0** | **2** | **0** | **0** | **1** | **0** | **1** | **0** | **2** | **0** | **0** |
| 5 | Vitamin E  (n = 1) | **1** | **0** | **0** | **0** | **1** | **0** | **0** | **0** | **1** | **0** | **0** | **1** | **0** | **0** | **1** | **0** | **0** | **0** | **1** | **0** | **0** |
|  | Placebo  (n = 1) | **1** | **0** | **0** | **0** | **1** | **0** | **0** | **0** | **1** | **0** | **0** | **1** | **0** | **0** | **0** | **0** | **1** | **0** | **1** | **0** | **0** |

Legend: n number of included cats; Score: 0‒4 mild clinical signs; 5‒7: moderate clinical signs; 8‒10: severe clinical signs; > 10: unacceptable clicical signs; euthanasia
