# Supplementary material for: Supplementation of vitamin E as an addition to a commercial renal diet does not prolong survival of cats with chronic kidney disease
Source: BMC Vet Res. 2024 Jul 10;20:308. doi: 10.1186/s12917-024-04176-8 (PMC11234628; doi:10.1186/s12917-024-04176-8)
Supplement: Supplementary file 3 — Supplementary Material 3 [file 12917_2024_4176_MOESM3_ESM.docx]

Supplementary Table 3: Key for the assessment of the severity of clinical signs

| Alertness (assessed by the owner) | Body weight loss | Dehydration | Appetite | Nausea, vomiting | Ulcerations in the oral cavity |
| --- | --- | --- | --- | --- | --- |
| **0** (very alert) | **0 (**≤5 %) | **0** (≤5 %) | **0** (normal) | **0** (not observed) | **0** (not present) |
| **1** (alert) | **1 (**≤10 %) | **1** (5 - 10 %) | **1** (decreased) | **1** (sporadic) | **1** (present) |
| **2** (moderately responsive) | **2** (≤15 %) | **2** (>10 %) | **2** (anorexia) | **2** (often) |  |
| **3** (non-responsive) | **3** (>15 %) |  |  |  |  |

Score: 0‒4 mild clinical signs; 5‒7 moderate clinical signs; 8‒10 severe clinical signs; >10 unacceptable clinical signs; euthanasia
